# Commentary: Correlation between Patent Foramen Ovale, Cerebral “Lesions” and Neuropsychometric Testing in Experienced Sports Divers: Does Diving Damage the Brain?

**DOI:** 10.3389/fpsyg.2016.01254

**Published:** 2016-08-24

**Authors:** Emmanuel Gempp

**Affiliations:** Diving and Hyperbaric Medicine, French Armed Forces Health ServiceToulon, France

**Keywords:** diving, long-term effects, persistent foramen ovale, brain MRI, cerebral embolism

After the careful reading of a recent and interesting article published by Balestra and Germonpré (2016) suggesting the lack of correlation between white-matter brain MRI hyperintensities, neuro-psychometric alterations, and the presence of persistent foramen ovale (PFO) screened by contrast transoesophageal echocardiography in recreational scuba divers, I would like to provide additional precisions and also discuss some of their findings.

The paper that referred to “Gempp et al. (2008)” is inadequate. The reader should rather consider the following reference: Gempp et al. (2010). In that study, we clearly showed that the number and size of cerebral MRI signal abnormalities in 34 military divers who strictly adhere to decompression procedures were closely related to the grade of right-to-left shunt determined by transcranial Doppler. Our results were also in line with the conclusions of 2 other radiological investigations, not cited by Balestra and Germonpré (2016) (Schwerzmann et al., 2001; Billinger et al., 2011), suggesting a potential causal relationship between focal cerebral hyperintensities and the presence of this anatomical predisposition (i.e., PFO). In their work, Balestra and Germonpré (2016) reported only 5 out of 42 divers having a total of 5 lesion-like hyperintense signals detected by brain MRI while nearly 64% of them (including 3 out of 5 divers with positive imaging) exhibited a PFO of varying importance. Neither the presence of PFO nor the existence of signal abnormalities were associated with an impairment of neuro-psychometric tests battery in this population. Although this outcome may be seen as a reassuring message for the scientific community, it should be interpreted with caution since this negative result is based on a very small number of MRI findings, hence making difficult to find a reliable correlation between the extent of radiological brain changes, the performance of the neuro-psychometric tests used, and the patency of PFO. In parallel, the authors did not mention, probably for publication deadline reasons, the results of a recent meta-analysis comprising 7 medium/high quality studies that revealed a highly significant increase in prevalence of cerebral hyperintense spots found with MRI in 98 out of 279 healthy divers (mainly professional) compared to 44 out of 232 non-diver controls (OR 2.65, 95% CI 1.71–4.39, *p* < 0.001; Connolly and Lee, 2015).

Anyway, the authors should be strongly encouraged and acknowledged for their excellent work reinforcing the notion that uneventful repeated diving exposure may have a deleterious impact on higher cognitive functions of scuba divers. Whether the hemodynamically relevance of PFO favors the development of long-term brain damage rather than diving alone remains, however, to be elucidated. A longitudinal follow-up study of brain MRI hyperintensities prevalence and neuro-psychometric alterations in relation with the presence of PFO in a carefully selected group of scuba divers could be the best way to make progress in this area.

## Author contributions

The author confirms being the sole contributor of this work and approved it for publication.

### Conflict of interest statement

The author declares that the research was conducted in the absence of any commercial or financial relationships that could be construed as a potential conflict of interest.
